# Evolution of Population Structure, Reproductive Performance, Inbreeding, and Genetic Diversity in Ecuadorian Charolais Cattle

**DOI:** 10.3390/vetsci11110566

**Published:** 2024-11-14

**Authors:** Luis F. Cartuche-Macas, Edwin F. Lozada, Miguel A. Gutiérrez-Reinoso, Edilberto Chacón, Francisco J. Navas, Manuel García-Herreros

**Affiliations:** 1 Instituto de Investigación de la Biodiversidad “Pachamamata Kamak”, Universidad Intercultural de las Nacionalidades y Pueblos Indígenas (UINPIAW), Quito 170524, Ecuador; 2 Asociación Charolais del Ecuador (ACE), Macas 140101, Ecuador; 3 Facultad de Ciencias Agropecuarias y Recursos Naturales, Carrera de Medicina Veterinaria, Universidad Técnica de Cotopaxi (UTC), Latacunga 050150, Ecuador; 4 Laboratorio de Biotecnología Animal, Departamento de Ciencia Animal, Facultad de Ciencias Veterinarias, Universidad de Concepción (UdeC), Chillán 3780000, Chile; 5 Dirección de Posgrado, Universidad Técnica de Cotopaxi (UTC), Latacunga 050150, Ecuador; 6 Departmento de Genética, Facultad de Veterinaria, Universidad de Córdoba (UCO), 14014 Córdoba, Spain; 7 Instituto Nacional de Investigação Agrária e Veterinária (INIAV), 2005-424 Santarém, Portugal; 8 CIISA-AL4AnimalS, Faculty of Veterinary Medicine, University of Lisbon, 1300-477 Lisbon, Portugal

**Keywords:** demographic structure, reproductive efficiency, inbreeding, genetic diversity loss, Charolais breed, beef cattle

## Abstract

The objective was to evaluate the evolution of the population structure, reproductive performance, inbreeding, and genetic diversity in Charolais cattle. Official genealogical information from the Ecuadorian Charolais Association was divided into five populations: (a) historical (total), (b) 2008–2012 (natural mating period), (c) 2013–2017 (natural mating + artificial insemination period), (d) 2018–2022 (artificial insemination + embryo transfer period), and (e) the reference (known sires and dams) from which the population structure was evaluated using pedigree completeness and the generation interval (GI). Meanwhile, for genetic diversity (GD), inbreeding (F), average relatedness (AR), and the effective population size (Ne) were estimated. The gene origin probability (number of founders, effective number of founders/ancestors, and number of founder genomes) explaining genetic variability, reproductive efficiency, the number of offspring per sire and dam, and the average ages of parents at the birth of their offspring were determined. The database was analyzed using the ENDOG, POPREP, and CFC software programs. The results indicate that pedigree completeness and the GI decreased over time among populations, although the first generation showed higher values. The sire gametic pathway was greater in all populations. F increased over time, although not markedly. Although Ne variations were observed, the levels remained above the recommended limit value. The GD loss increased progressively over time although the most recent population was not significantly affected. Finally, the female reproductive efficiency increased over time as well. In conclusion, the Charolais cattle GI should be reduced. After 2012, the use of Assisted Reproductive Technologies and breeding schemes raised the GD loss. Finally, the F increased over time although it could be reduced using foreign genetic resources in the current Ecuadorian Charolais population.

## Simple Summary

Studies on demography, reproductive traits, and genetic diversity in Charolais cattle are scarce in Ecuador. The evolution of the population structure, reproductive performance, inbreeding, and diversity loss were analyzed through official pedigree records based on the two types of existing herd books in Ecuadorian Charolais cattle as part of a model for application in other cattle breeds. The results from our study showed that the application of different breeding methods using genetically top-rated individuals influenced the inbreeding rates and genetic diversity loss over time, which could be minimized using foreign genetic resources in the current Ecuadorian Charolais cattle population.

## 1. Introduction

The Charolais is a cattle beef breed that originated in central France (region: Charolles). This cattle breed expanded its area of distribution in South America during the second half of the 20th century, including in Ecuador, whose beef industry began to expand. The establishment of the Charolais cattle breed in Ecuador went through three stages. The first stage began at the end of the 1960s, when the first individuals arrived in Ecuador, specifically in the Upano Valley, Macas, and Morona Santiago [1]. The second stage began in 1996 with the importation of purebred cattle from the United States [1]. Finally, the third stage began in the 2000s with the use of different assisted reproductive technologies such as artificial insemination and embryo transfer using genetic material from purebred Charolais cattle from France. Currently, this breed has been formally established due to its successful adaptation; however, it was necessary to create the Ecuadorian Charolais Association in order to guarantee the genetic quality of cattle through genealogical records increasing the performance in terms of beef production. Thus, the Charolais Association began with 20 initial herds including animals born since 2008. Within the process of the establishment and genetic management of the Charolais breed, two herd books were created. The first one was called “Pure French” or “Full French” and included only animals with DNA parentage proofs guaranteeing their genetic origins. The second was called “Pure Bred”, including animals obtained from crosses from the initial I-population (unknown parents). Thus, from the initial population, the first generation was generated (known parents), then the second (known parents and grandparents), third (known parents, grandparents, great grandparents), and fourth (known parents, grandparents, great grandparents, and great-great grandparents). Finally, only the fifth (purity: 31/32) could be registered in the PB herd book. The processes of establishing improved breeds in the different countries have been carried out through the use of ARTs such as AI and ET, which implies the intensive use of a small group of genetically superior sires and cows from different origins. In Ecuador, the origin of the Charolais cattle breed was mainly French. Thus, the application of ARTs could lead to genetic changes in the population structure and genetic diversity, as well as increased inbreeding, which can result in inbreeding depression [2,3] and increase the number of deleterious genes within the population [4,5]. This is important because low genetic diversity (limited variety of alleles for genes within a breed) means that there are not many differences among individuals, and therefore, there are fewer opportunities to adapt to environmental changes.

Genealogical information allows to analyze and monitor the genetic structures of populations [6,7], as well as genetic diversity [8,9,10,11], the gene origin probability based on founders [12,13], ancestors [14], and their different ancestral contributions [15]. Worldwide, there are several methodologies that have been used to monitor some populations of individuals in different cattle breeds [16,17,18,19,20,21]. These methodologies are based on the use of specialized analysis software such as ENDOG [22], CFC [23], POPREP [24], EVA [25], and PEDIG [26], among others.

Thus, after approximately two decades since the creation of the initial genealogical records in Ecuador, studies on the population structure and genetic diversity of the Charolais cattle breed have been scarce [1]. Then, the main objective of the present study was to determine the evolution of the population structure, reproductive performance, inbreeding, and genetic diversity of Ecuadorian Charolais cattle through analyzing official pedigree records based on the two types of existing herd books and by analyzing the groups of animals obtained through the use of different breeding methods [natural mating, artificial insemination, and embryo transfer].

## 2. Materials and Methods

### 2.1. Ethical Statement

The present research did not require any animal handling since the study was directly carried out using the records and databases provided by the Institut de l’élevage (IDELE, France), the Irish Cattle Breeding Federation (ICBF, Ireland), and the Ecuadorian Charolais Association (ECA, Ecuador).

### 2.2. Charolais Genealogical Database

The genealogical information was obtained from the database of the software Intertrace v. 1.1.0, which is used to manage the genealogical records of the Ecuadorian Charolais Association (Ecuador). This database is made up of animals born in Ecuador from the beginning of 1999 until December 2022. The database included the following: individual identification, sire and dam identification, sex, date of birth, reproductive origin (NM, AI, or ET), and geographical location. In the case of imported animals, the pedigree information was obtained from international databases of the Institut de l’élevage (IDELE, France) and the Irish Cattle Breeding Federation (ICBF, Ireland). The database consisted of 4961 animals, of which 1427 were males (28.76%) and 3534 females (71.24%). The animal distribution was 84.48% from Ecuador, 13.53% from France, 1.87% from the USA, and 0.12% from other countries.

Pedigree information was divided into four groups: (a) historical population (the whole population), (b) population of individual born between 2008 and 2012 (beginning of herd book), (c) born between 2013 and 2017, and (d) born between 2018 and 2022, according to the methodology of Navas et al. [27]. For the analysis of genetic diversity and probability of gene origin, an additional reference database was constructed with individuals with both parents known within the 2018–2022 population. In addition, analyses were performed on both the type of book—Full French (FF) and Purebred (PB)—as well as the breeding method used (NM, AI, or ET).

### 2.3. Demographic Analysis

The demographic analysis was carried out as follows:

1. Demographic distribution: Number of females per number of births, number of males and females, and average age of offspring per year were used as criteria to determine the population structure in subsequent generations.
2. Pedigree completeness: It was analyzed through the known ancestors up to 5 generations of individuals born in the proposed periods according to MacCluer et al. [28]. In addition, the numbers of complete (GCom), maximum (GMax), and equivalent (GEqu) generations were considered. In the case of GEqu, the following formula was used:
  EqGi=∑12n
  Here, “*n*” is the number of generations in which the individual is separated from each ancestor with a known record. The pedigree completeness was analyzed through the numbers of GEqu, GMax, and GCom [29] and the pedigree completeness index [28].
3. Generation interval (GI): The GI was defined as the average age of the parents at the birth of their offspring. The GI was calculated for the 4 gametic pathways: Sire–Son, Sire–Daughter, Dam–Son, and Dam–Daughter [6].

### 2.4. Reproductive-Performance-Derived Parameters

The evolution of the number of registered animals by type of Assisted Reproductive Technology (ART) used in the Ecuadorian Charolais population was studied by dividing the whole period (2008–2022) into three periods as follows: (a) 2008–2012 (when natural mating was predominant), (b) 2013–2017 (when artificial insemination was predominant), and (c) 2018–2022 (when artificial insemination and embryo transfer were predominant). Based on the historical official records obtained from the Ecuadorian Charolais Association, several parameters related to the reproductive performance were analyzed during the different chronological periods. The number of births obtained from the official records was analyzed to establish both the number of offspring per sire and dam as follows: (a) the average number of calves per sire, (b) the maximum number of calves per sire, (c) the average number of calves per dam, and (d) the maximum number of calves per dam. Moreover, the average age of parents at the birth of offspring and generation intervals were recorded and analyzed using birth date records for each individual and their respective parents (sire and dam). In addition, the distribution of cows by calving number (calves/cow/year) was obtained. Finally, the influence of the type of ART used based on the different chronological periods studied was analyzed for inbreeding, relatedness, coancestry, non-random mating, and genetic conservation parameters, as well as for gene origin and genetic-diversity-derived parameters.

### 2.5. Inbreeding, Relatedness, Coancestry, Non-Random Mating, and Genetic Conservation Parameters

Inbreeding coefficient (F): The F was estimated using the algorithm proposed by Meuwissen and Luo [30] and the inbreeding increment (ΔF) per generation was calculated using the equation proposed by Gutiérrez et al. [10]:

∆F=Ft−Ft−11−Ft−1

Here, *F_t_* and *F_t_*_−1_: average inbreeding of the tth generation (i = 1, …, *t*).

Average relatedness (AR): AR was defined as the probability that a randomly selected allele from a population belonged to a specific individual, which was calculated using the vector c, where each element corresponded to the respective AR of an individual, defined by Gutiérrez and Goyache [22]:

C′ = (1/n)1′A

Here, “A” is the n × n parentage matrix.

Coancestry (C): The C between two individuals is equal to the inbreeding coefficient of their offspring if the individuals are related [8]. It was also used to analyze the degree of relatedness and non-random mating, α, within breeds. The coancestry was estimated according to the algorithm of Colleau [31].

Non-random mating (α): The α was estimated as the correlation of genes between two individuals in relation to the correlation of genes taken at random from the population (α) according to Caballero and Toro [32]. It indicates the degree of deviation from Hardy–Weinberg proportions and is related to the inbreeding coefficients according to Sheppard and Wright [33]:

$$\left(1-F\right)=\left(1-C\right)\left(1-\alpha \right)$$

Effective size (Ne): The Ne was defined as the number of males and females contributing to genetic variability in a population [34]. It was calculated as proposed by Hill [35]:

Ne=12∆F

Three additional Ne values were also estimated using the regression coefficient (b) of the individual inbreeding coefficients on (i) the full number of generations, (ii) the maximum number of generations, and (iii) the equivalent number of full generations, with the regression coefficient corresponding to the increment between the two inbreeding generations (Fn − Fn_−1_ = b) [34]:

Ne=12b

Genetic Conservation Index (GCI): The GCI was estimated from the genetic contribution of all founders, considering the proportion of genes from a founder animal in the pedigree under analysis according to Alderson [36]. The following equation was used:

GCI=1∑pi2

Here, “*p_i_*” is the proportion of genes of founder “*i*” in the individual’s pedigree.

### 2.6. Gene Origin and Genetic-Diversity-Derived Parameters

Number of founders: The number of founders was defined by including those individuals with unknown parents assumed to be unrelated and with an inbreeding coefficient of 0.

Effective number of founders (fe): The fe was defined as the number of founders that contributed equally and were expected to produce the same genetic diversity as the study population [13]. It was estimated from the following equation:

fe=1∑k=1fqk2

Here, “*q_k_*” is the gene origin probability from ancestor “*k*”.

Effective number of ancestors (*f_a_*): The fa was defined as the minimum number of ancestors that were not necessarily founders and that accounted for the full genetic diversity of a population according to [13]:

fa=1∑j=1aqj2

Here, “*q_j_*” is the marginal contribution of an ancestor “*j*” that is not explained by other chosen ancestors.

Number of founder genome equivalents (fg): The fg was defined as the number of founders that would be expected to produce the same genetic diversity as the population under study if the founders were equally represented and no allele loss occurred. This was estimated from twice the inverse of the average C according to Caballero and Toro [32].

Fe/fa and fg/fa ratios were estimated to determine genetic bottlenecks and random genetic drift, respectively.

Genetic contributions: The genetic contributions were estimated for the top ten ancestors with the maximum genetic impact between 2008 and 2022.

Genetic diversity (GD): The GD was estimated from the following equation:

GD=1−12fg.

Genetic diversity loss (GD loss): The population GD loss from the founder generation was estimated using 1 − GD. The GD loss due to unequal contribution of founders was estimated according to Caballero and Toro [32] using 1 − GD*:

GD*=1−12fe

The unequal contribution of founders relates to the fact that the genetic contributions of founders of specific populations may be of different proportions due to past directional mating (human-mediated or not) during the process of population shaping. The difference between GD and GD* indicates the GD loss due to genetic drift accumulated from the population founding [13] and the effective number of non-founders (Nenf).

### 2.7. Data Analysis and Software

The software programs used for the database analysis were ENDOG v. 4.8 [22], POPREP [24], and CFC [23], by means of which the demographic-derived parameters, genetic diversity indices, and gene origin probability were obtained.

## 3. Results

### 3.1. Evolution of Population Structure and Reproductive Performance in Ecuadorian Charolais Cattle

#### 3.1.1. Demographic Structure and Reproductive Performance (Calving Rates)

Table 1 shows the evolution of demographic structure and reproductive performance in Charolais cattle in Ecuador. Despite a peak in the number of animals belonging to the pedigree in the five-year period 2013–2017, the general trend from the first five-year period studied (2008–2012) and the last five-year period (2018–2022) was a decrease (15.71%) in the number of animals belonging to the pedigree. Regarding the number of animals in the reference population, an increase of 45.40% was observed between the five-year periods 2008–2012 and 2018–2022. However, if the five-year period 2013–2017 was compared to the five-year period 2018–2022, the reference population decreased by 20.25%. Overall, the total number of parents increased from the first five-year period to the last five-year period. However, the trend in the last five-year period compared to the previous one was to decrease the total numbers of sires and dams by 15.28% and 32.35%, respectively. Regarding the number of individuals with and without progeny, a decrease of 81.12% in the number of individuals with progeny was observed between the first and last five-year periods while an increase of 30.53% in the number of individuals without progeny was observed. In general, the numbers of animals with only with a known sire, a known dam, and no known (both) parents were also considerably reduced from the five-year period 2008–2012 to the five-year period 2018–2022. For example, for the five-year period 2018–2022, the number of animals only with known dams was just two, compared to fifteen in the 2008–2012 period. On the other hand, the number of animals only with known sires decreased from eighty-eight (first five-year period studied) to six (last five-year period), with the vast majority of individuals belonging to the group of animals with no known parents, which decreased from 617 (2008–2012 period) to 59 (2018–2022 period).

When analyzing the average numbers of calves (offspring) per sire and dam, the average numbers decreased drastically from the five-year period 2008–2012 to the five-year period 2018–2022 by 1.74 (23.1%) and 0.27 (12.7%), respectively. Similarly, the maximum numbers of calves per dam and sire decreased from thirteen to eight (38.4%) and from forty to twenty-four (40.0%), respectively.

#### 3.1.2. Breeding Sires’ and Dams’ Average Ages

**Figure 1:**
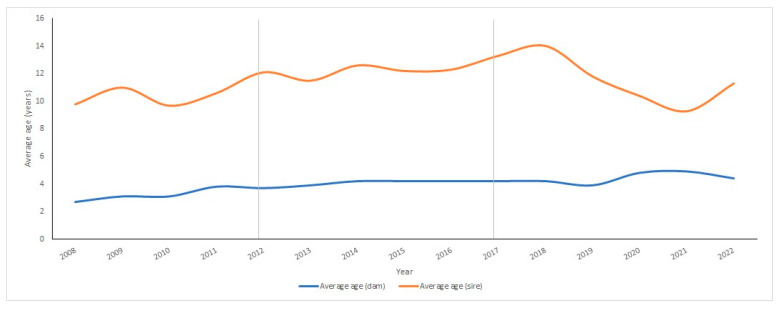
Average ages of breeding sires and dams in the Ecuadorian Charolais cattle population (2008–2022).

Figure 1 shows the evolution of the average ages of breeding sires and dams per year.

In the case of sires, the age increased from 9.8 years (2008) to 11.30 years (2022), while in the case of dams, it increased from 2.7 years (2008) to 4.4 years (2022) (Figure 1).

#### 3.1.3. Evolution of the Use of ARTs in the Ecuadorian Charolais Cattle Population from 2008 to 2022

**Figure 2:**
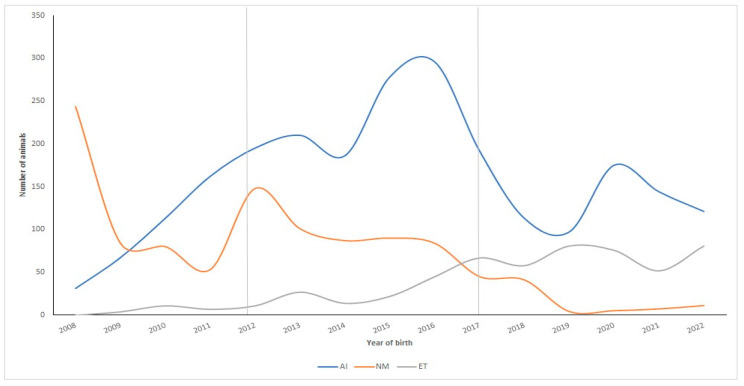
Evolution of the numbers of registered animals by type of Assisted Reproductive Technology (ART) used in the Ecuadorian Charolais population (2008–2022). NM: natural mating; AI: artificial insemination; ET: embryo transfer.

Figure 2 shows the evolution of the numbers of registered animals by the type of Assisted Reproductive Technology in the Ecuadorian Charolais cattle population over time (2008–2022).

Artificial insemination (AI) was the most used reproductive ART over time, although in recent years, it has had a downward trend. AI was followed by ET, which had a progressive upward trend (2013–2022), reaching, during the last period (2018–2022), the highest record ever in the history of the Charolais cattle breed in Ecuador compared to the other chronological periods. Finally, the use of NM was relegated to a very low number of animals, remaining marginal compared to 2008, when it was the most used reproductive practice (Figure 2).

#### 3.1.4. Number of Calves Obtained per Cow from 2008 to 2022

**Figure 3:**
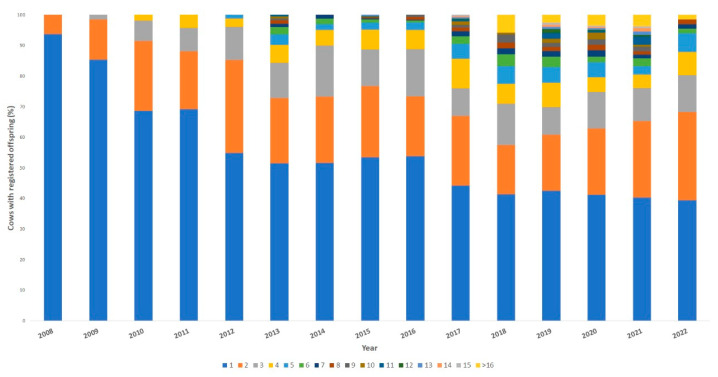
Distribution of dams by the number of offspring (calves/cow/year) from 2008 to 2022. Over the years, the percentage of cows with one calving (blue: a calf per cow/year) decreased drastically while the percentages of cows with two (orange), three (grey), and four (yellow) calves per year increased significantly over time, especially in the last two five-year periods analyzed.

Figure 3 shows the distribution of the % of dams by the number of offspring (offspring/cow/year) in the Ecuadorian Charolais cattle population from 2008 to 2022

From the last five-year period (2018 onwards), there were numerous animals showing more than four calves per cow/year, coinciding with the implementation of ET technologies on a massive scale in this breed (Figure 2).

### 3.2. Evolution of Pedigree Completeness and Generation Intervals in Ecuadorian Charolais Cattle

#### 3.2.1. Pedigree Completeness in the Ecuadorian Charolais Cattle Population from 2008 to 2022

Table 2 shows the pedigree-completeness-derived parameters in Ecuadorian Charolais cattle from 2008 to 2022. Overall, the pedigree completeness increased significantly between 2008 and 2022. Depending on the period analyzed, sometimes, the completeness in the first generation in each period was almost double that of the second generation, and in turn, the completeness of the third generation could be three times higher than that of the fourth generation. Similarly, there was a 28–48% reduction in the number of registered animals in the 2018–2022 period was compared to the 2008–2012 period.

The difference between GCom and GMax analyzed between 2018 and 2022 in the Ecuadorian Charolais cattle increased significantly compared to the previous five-year periods. In the same way, it was observed that the differences between the GCom and GMax of the FF herd book were greater than those of the PB herd book.

#### 3.2.2. Generation Intervals in Ecuadorian Charolais Cattle Population from 2008 to 2022

**Figure 4:**
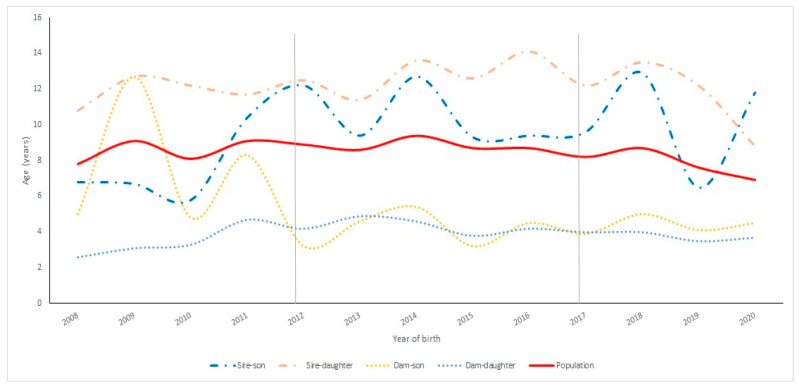
Evolution of the generation interval (GI) regarding the different gametic pathways in Ecuadorian Charolais cattle from 2008 to 2022. Data from before 2008 were excluded because the Charolais Association began in 2008, including animals born and registered that year onwards.

Figure 4 shows the evolution of the GI regarding the different gametic pathways in Ecuadorian Charolais cattle. In general, there was a tendency for the GI of the population to remain stable over time. Gradual increase–decrease variations in GI values were observed for the Ecuadorian Charolais breed population in all influential gametic pathways (Sire–Son, Sire–Daughter, Dam–Son, and Dam–Daughter) between 2008 and 2022. The GI fluctuations were minimal in the Dam–Son and Dam–Daughter gametic pathways, especially during the second and third chronological period (2013 onwards, corresponding to the NM + AI and AI + ET periods, respectively). The most accentuated fluctuations in the GI value were observed in the Sire–Son and Sire–Daughter gametic pathways over time; however, just about a couple decades after the introduction of ET technologies, GI values fluctuated significantly in the male Sire–Son gametic pathway whereas in the Sire–Daughter gametic pathway, a progressive decrease was observed regarding the GI value.

It is important to note that accentuated differences regarding the GI were observed between male and female gametic pathways over time; however, during the NM period (2008 to 2012), the Dam–Son pathway was above the Sire–Son gametic pathway. Then, the GI related to the Dam–Son pathway decreased drastically until the beginning of the NM + AI period (2012) onwards, maintaining a stable GI value until 2022. Finally, the GI value related to the Sire–Son pathway was triggered during the last years (2019 onwards), exceeding the GI value related to the Sire–Daughter pathway for the first time since 2008 (Figure 4).

#### 3.2.3. Inbreeding, Average Relatedness, Coancestry, and Non-Random Mating in Ecuadorian Charolais Cattle Population from 2008 to 2022

**Figure 5:**
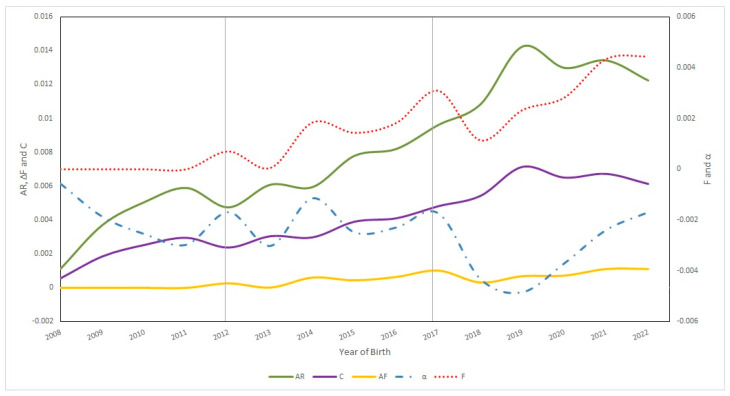
Evolution of inbreeding (F and ΔF), average relatedness (AR), coancestry (C), and non-random mating (α) in Ecuadorian Charolais cattle population from 2008 to 2022. Data from before 2008 were excluded because the Charolais Association began in 2008, including animals born and registered that year onwards.

Figure 5 shows the evolution of inbreeding, average relatedness, coancestry, and non-random mating in the Ecuadorian Charolais cattle population from 2008 to 2022.

Between 2013 and 2022, a significant increase in the inbreeding coefficient (F) was observed among the different periods. Between 2018 and 2022, the average value of F was 0.33 ± 1.37%, with an individual increase in the F of 0.08 ± 0.34 and a maximum inbreeding of 25%. The population of animals with some degree of inbreeding increased in the last period with 27.84%; however, despite the fact that in the last period, the F values in the different genealogical parameters increased, in 2022, the coancestry and average relatedness values decreased whereas the F and ΔF remained stable and the non-random mating (α) values tended to increase. Figure 5 also shows an increase in the number of inbred individuals in FF individuals while the number of inbred PB animals tended to decrease. Even so, the levels of inbreeding in PB individuals presented a strong increase in F since 2018, while in the case of FF individuals, it tended to increase moderately since 2021.

Regarding the genetic conservation index (GCI), lower values were observed in the first two periods studied (2.19 ± 1.59 vs. 3.86 ± 2.70 for the 2008–2012 and 2013–2017 periods, respectively); however, in the third period (2018–2022), an increase of almost double (GCI: 7.18 ± 3.20) that obtained in the 2013–2017 period was observed.

**Figure 6:**
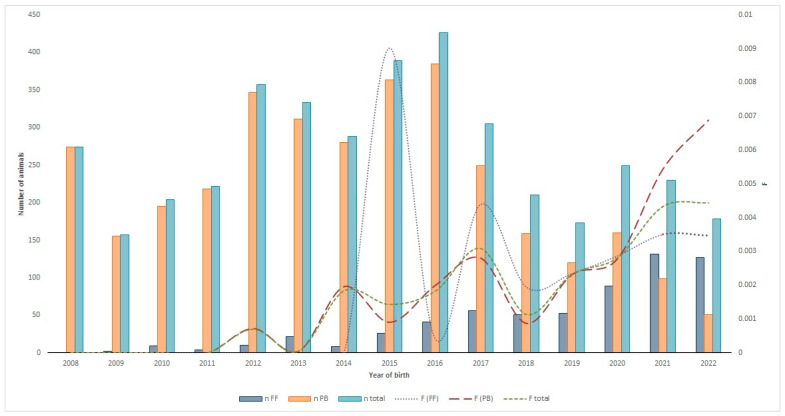
Evolution of Ecuadorian Charolais breed belonging to FF and PB herd books regarding the total animal population and inbreeding rate. Data from before 2008 were excluded because the Charolais Association began in 2008, including animals born and registered that year onwards.

Figure 6 shows the evolution of the Charolais cattle breed belonging to the FF and PB herd books regarding the total population and inbreeding coefficient (F) from 2008 to 2022. The results showed that the proportion of inbred individuals of the Ecuadorian Charolais breed had increased over time, irrespective of the herd book considered. Between 2013 and 2017 (NM + AI period), the proportion of inbred individuals was significantly higher in animals registered in the FF herd book compared to the other two chronological periods (Figure 6); however, although the proportion of inbreeding in the last two analyzed periods was high, the mean inbreeding coefficient (F total) value was quite moderate (0.0045). It is worth noting that during the last five-year period (2018–2022), the F coefficient was triggered in individuals registered in the PB herd book. This fact reveals that there were important changes in breeding management schemes that had had a huge influence on the inbreeding coefficient values during the last five-year period (AI + ET period).

Regarding the evolution of the total population of the Ecuadorian Charolais breed, a significant increase was observed during the second chronological period (2012–2017) with the large-scale implementation of AI technologies, especially in the individuals belonging to the FB herd book (Figure 6). After the second chronological period, the population of individuals registered in the PB herd book diminished over time. On the contrary, the individuals registered in the FF herd book increased progressively, and was more accentuated in the last chronological period (2018–2022), reaching the highest population ever in the history of the Charolais FF herd book, coinciding with the implementation of ET technologies on a massive scale. In fact, for the first time in the history of the Charolais cattle breed in Ecuador, the individuals registered in the FF herd book had exceeded the number compared to those in the PB herd book.

#### 3.2.4. Effective Size (Ne) in Ecuadorian Charolais Cattle Population from 2008 to 2022

**Figure 7:**
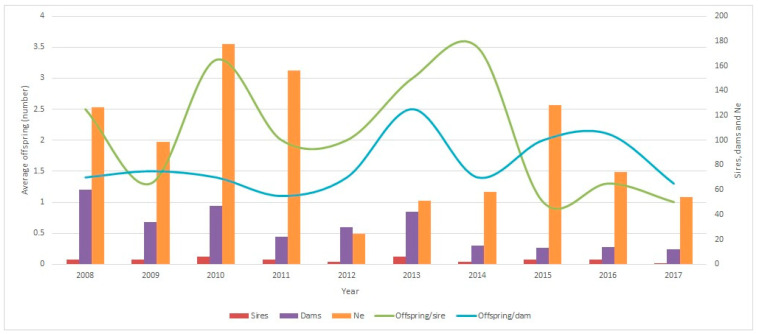
Evolution of the effective size (Ne) in Ecuadorian Charolais cattle population. Data from before 2008 were excluded because the Charolais Association began in 2008, including animals born and registered that year onwards.

The effective size (Ne) of the Ecuadorian Charolais cattle population (per year) was quite unstable until 2015, varying from 18.39 (2008) to 155.58 (2010). On the other hand, the Ne value between 2015 and 2017 decreased from 84.99 to 53.69, as shown in Figure 7. In general, a slight increase in the Ne was observed from 2008 to 2017 (first and second periods). Due to the lack of ancestors to calculate the Ne, the last period (2018–2022) has not been included in Figure 7.

Additionally, the Ne values using the regression coefficient (b) of the individual inbreeding coefficients (F) resulted in a complete number of generations (GCom) of 235.04, and the maximum number of generations (GMax) was 1281.96. Finally, the equivalent number of full generations (GEqu) was 436.94.

### 3.3. Evolution of the Gene Origin Probability-Derived Parameters in Ecuadorian Charolais Cattle

#### 3.3.1. Gene Origin Probability in Ecuadorian Charolais Cattle Population from 2008 to 2022

A summary of all parameters derived from the gene origin and genetic diversity analysis in the different chronological periods is shown in Table 3. Among the studied periods, the highest number of founders contributing to the reference population (n) was detected between 2013 and 2017 (847); however, between 2008 and 2012 (the period when NM was predominant), a lower number of founders was observed (539). Similarly, the same trend also was observed for the effective number of founders (fe) and the founder genome equivalents (fg). The highest values of fe and fg were reached between 2013 and 2017 (period when NM + AI was predominant) (fe: 189.65; fg: 61.33) and between 2008 and 2012 (period when NM was predominant) (fe: 168.92; fg: 61.19), respectively, and the lowest values for fe and fg were found between 2018 and 2022 (period when AI +ET was predominant) (fe: 131.81; fg: 29.03). Overall, after the occurrence of the AI + ET in the genomic era (2013 onwards), the fe and fg of the Charolais cattle breed decreased. When different chronological periods were compared, the fa/fe and fg/fe ratio showed a similar trend between 2008 and 2017 (periods when NM and NM + AI were predominant); however, these ratios were lower between 2018 and 2022 (AI + ET period). Overall, both the fa/fe and fg/fe ratios decreased over time (Table 3).

#### 3.3.2. Genetic Diversity in Ecuadorian Charolais Cattle Population from 2008 to 2022

A summary of genetic-diversity-derived parameters during the different chronological periods is shown in Table 3. The main cause of GD loss in Ecuadorian Charolais cattle breed was shown to be genetic drift accumulated over non-founder generations, which was mainly caused by a small Ne, irrespective of the chronological period studied. Moreover, the unequal contribution of founders and bottlenecks jointly contributed relatively equally to the total loss during 2008–2012 (the NM period) and 2013–2017 (the NM + AI period). Overall, the GD loss increased over time, being the highest during the 2018–2022 period (Table 3).

## 4. Discussion

In the present study, the evolution of the population structure, reproductive performance, inbreeding, and genetic diversity loss in Ecuadorian Charolais cattle was analyzed over time from 2008 to 2022. Overall, the evolution of the different parameters studied was quite similar to those obtained in other South American countries where the Charolais breed is being established [37]. Genetically, the Ecuadorian Charolais cattle is a population that remains in an initial phase of expansion, although the results obtained in recent years have shown a reduction in the population of this breed [1]. For example, a reduction in the population structure was observed in the last five-year period studied (2018–2022), possibly due to the effect of the economic and health crisis that occurred in Ecuador during the years 2020–2022, which meant that the number of animals registered was only a third of that registered between 2013 and 2017. This fact is part of the initial establishment process when a breed arrives recently to a country, as has happened in other countries with other different beef breeds such as the Braford breed in Argentina [38], showing lower pedigree completeness values in the populations studied. Therefore, in the present study, an analysis was carried out from 2008 onwards, when the herd book was formed using only registered animals [1]. On the other hand, the population values of animals with only a known sire, only a known dam, and both unknown parents were significantly reduced, possibly due to an effect derived from the improvement of both pedigree completeness as well as other parameters related to genetic diversity.

In other breeds, such as the Polish red breed, higher values have been observed regarding different parameters, such as the average family size [39], whose value is higher than in the case of the Charolais breed developed in Ecuador. With respect to the evolution of the average age of animal breeders (sires and dams), an increase over time in both males and females was observed. This effect indicates that the female population was increasing its longevity over time, although it could have also been due to a possible effect derived from the use of older females in embryo transfer processes due to their belonging to the genetic elite group among the entire existing population [40]. The evolution of the GI in the Charolais cattle population showed a tendency to increase over time, increasing until 2022 with values higher than those obtained for the same breed in other European countries such as Denmark, France, Ireland, and Sweden [41] and in America, such as in Mexico [42]. On the other hand, the GI values for the Charolais cattle breed in Italy between 1960 and 2017 were 6.30 ± 7.29 years for the Sire–Son pathway, 5.44 ± 4.7 for the father–daughter pathway, 7.57 ± 8.5 for the mother–son pathway, and 7.81 ± 8.18 for the mother–daughter pathway, with a total average of 6.70 ± 7.01 years [37]. These values differed slightly from the values obtained for the same breed developed in Ecuador. On the other hand, the GI increased in the sire–daughter pathway, which was associated with the use of sires with a high degree of age variation when their offspring were born, as is the case with the Charolais breed in Mexico [42], which has also occurred in other countries with other breeds [43].

With regard to the pedigree completeness, a significant improvement was observed between 2008 and 2022. This effect was possibly due to improved animal registration processes (e.g., in the FF book, where molecular genetic technologies have been used for parentage verification). Thus, obtaining the difference between the GCom and GMax values, then, the pedigree completeness was verified. For instance, closer values between these two parameters indicate a pedigree with few unknown animals [44]. In the case of the Charolais breed analyzed between 2018 and 2022, a significant increase in the difference between GCom and GMax was observed with respect to the previous five-year periods. This could indicate the presence of unknown animals in the population (e.g., 59 animals with unknown sires and dams were recorded between 2018 and 2022). These results were similar to the Italian Charolais population obtained between 1960 and 2017, and also between 1980 and 2018, reported by de Rezende et al. [37] and Fabbri et al. [20], respectively. In the same way, it was observed that the differences in the FF herd book were higher than in the PB book. On the other hand, an increase in the GEqu value was observed in both herd books, although to a lesser extent in the FF herd book. This trend has also been observed in the Charolais population of Mexico with higher values over time in individuals born between 1984 and 2018 [42] and in the European Charolais populations of Denmark with 8.5, France with 9.3, Ireland with 9.1, and Sweden with 8.3 [41]. This fact may be due to the depth of the pedigree in these populations during the first three generations, which presented values higher than 90% for known ancestors, while in the Ecuadorian population, only the two generations belonging to the five-year period 2018–2022 exceeded 80% for known ancestors. When comparing the GEqu values of the FF and PB herd books between the Italian and Ecuadorian Charolais populations, the values were quite similar for the PB and FF herd books, respectively [20,36]. This could have been due to the fact that the values of pedigree completeness and pedigree depth in the populations between the first and third generation were lower than 77.27%, which was similar to what was found in the Ecuadorian population.

The rate of genetic progress in the Charolais breed population depends, among other things, on the turnover of animals selected for breeding [45]. In general, individuals that stay longer in the population tend to leave more offspring because their reproductive activity has been spread over time. Therefore, the distribution of dam parity over time may be a determining and influential factor underlying the turnover rates of individuals selected for breeding in the whole population [24]. Regarding the evolution of the use of reproductive biotechnologies in the Ecuadorian Charolais population, it has been observed that AI has been the most used ART to date, followed by ET. This fact could generate important genetic gains; however, it could also accentuate the deterioration of genetic diversity due to a possible reduction in the generation interval considerably [46] with a consequent potential increase in inbreeding rates. In the present study, such an effect would possibly not have affected the Charolais population as there was a tendency to increase the GI, especially in the paternal pathway and slightly also in the maternal pathway. Additionally, it has been observed that in some cattle populations, the increased use of ARTs and genomic selection technologies has directly influenced cattle selection and breeding programs, affecting genetic diversity in an accelerated manner [47]. Because of this, it would be necessary to consider the relatedness of selected sires and dams in the near future, given that information from the first genomic tests conducted on Charolais females in Ecuador is still in the process of being obtained.

Regarding the evolution of the inbreeding-derived parameters of the populations in the present study, a significant increase in the inbreeding coefficient (F) was observed among the different chronological periods analyzed. Thus, the F value obtained between 2013 and 2022 (last two five-year periods) doubled, contrary to what happened with the Charolais population of Mexico, which has maintained a constant stability since 1992 [42]. This may be due to the fact that in Mexico, between 2000 and 2018, a greater number of founder animals was recorded. In addition, it could also have been due to the crossing of national individuals with imported sires, while in Ecuador, the number of animals registered without known fathers or with only a known sire decreased. The GCI values increased over time and indicated that there was no introduction of new germplasm from foreign countries in the nucleus population of Ecuadorian Charolais cattle. The GCI value expresses the importance of the conservation of the entire spectrum of alleles that belongs to the standard population. Generally, all individuals in the population would receive equal genetic contributions from the original ancestors, resulting in greater GCI values. Therefore, a greater GCI value means that an individual is much more valuable in terms of the conservation index. In the last period (2018–2022), the GCI value of Charolais cattle was greater compared to the other periods, and therefore, this indicated that the GCI value was a parameter to be taken into account to perform correct mating for future breeding and selection programs involving Ecuadorian Charolais cattle. Regarding the Ne value per year obtained in the Ecuadorian Charolais cattle population, which was rather unstable, a marked increase was observed between 2008 and 2013, while between 2015 and 2017, just a slight reduction was observed. The incremental effect was similar to that obtained in the Mexican Charolais population during the period between 1999 and 2013 [42]. According to recommendations for conservation programs, the Ne value should be between 50 and 100 as at this level, the inbreeding rate is 1% per generation [30,47]. In the present study, in the case of the Ecuadorian Charolais population, the Ne value was maintained within this range, which kept the Charolais population out of danger.

The gene origin probability of the Ecuadorian Charolais population in terms of the number of equivalent founders was lower than that reported for the Charolais population in Mexico between 2014 and 2018 [41]. Furthermore, in France, the first 10 ancestors contributed 19.2% of the genetic diversity for the population of Charolais females born between 2018 and 2021 [48], while in Ecuador, this value increased until 36.94%. However, no matching animals were observed as, in France, the ancestors were born between 1968 and 2001, while in Ecuador, they were born between 1994 and 2013. When analyzing the ancestry in Ecuador, it was determined that the sire Jumper (second ancestor in the ranking), the sire Artois (third ancestor in the ranking), and the dam BZJ Anabely 189 (fifth ancestor in the ranking) came from the sire Flambeau-FR0370119503 (1970), which was the second sire that contributed the most in terms of the GD in France with 2.6%. On the other hand, the sire Novotel (fifth ancestor) came from Durandal-FR2168105211 (1968), which was the 10th sire, contributing 1.4% to the GD of the Charolais population. Finally, the sire Natur (ninth ancestor in the ranking) came from the sire Echo-FR5869101650 (1969), which was the third most important sire in terms of the GD value, contributing 2.2% [48]. In contrast, in the Mexican Charolais population born between 2014 and 2018, only Jumper (eighth in the ranking) contributed 1% to the GD [42], and this sire was only matched by the Ecuadorian population, which contributed 5.79%. On the other hand, the sire Flambeau was the fifth ancestor with the highest contribution in the Mexican Charolais population but never appeared as an ancestor in Ecuador. The study by Lozada et al. [1] analyzed a much more restricted reference population (born between 2012 and 2021) and observed that the most influencing sires were Castor (first ancestor in the ranking), Jumper (second ancestor in the ranking), Artois (third ancestor in the ranking), Suedois (fifth ancestor in the ranking), and Novotel (sixth ancestor in the ranking); however, the sire Utopique did not appear as an ancestor in that study. In addition, within this study appeared several dams born in Ecuador as TK Nick 03 ET (fourth ancestor in the ranking) and BZJ Anabely 189 (fifth ancestor in the ranking), born in 2013, which represented 6.08% of the GD while, in France, they only represented 3.3% of the GD [48]. Finally, in the Charolais population analyzed by Lozada et al. [1], only the dam appeared, which contributed 1.05% to the GD.

## 5. Conclusions

In summary, based on the information derived from the progeny tests (FF and PB herd books), the Ecuadorian Charolais cattle should be managed to reduce the GI. The genetic-diversity-derived values showed that the application of ARTs (specifically AI and ET) and genomic selection schemes increased the GD loss in the Ecuadorian Charolais cattle. Currently, it should be a priority to control the inbreeding rates (F and ΔF) by increasing the Ne value of Charolais sires and dams in Ecuador. Finally, the ΔF has been increased over time, which could be controlled using foreign genetic resources to increase the GD in the current Ecuadorian Charolais population.

## Figures and Tables

**Table 1:** Demographic structure and reproductive performance parameters in Ecuadorian Charolais cattle from 2008 to 2022.

**Demographic Parameter**	Historical Period *	**2008–2012 Period**	**2013–2017 Period**	**2018–2022 Period**
Number of animals with pedigree	4961	1266	1781	1067
Number of animals (reference population)	3098	546	1254	1000
Sires (total)	470	105	157	133
Dams (total)	1593	356	717	485
Individuals with progeny (offspring)	2063	604	554	114
Individuals without progeny (offspring)	2898	662	1237	953
Number of animals with both known parents	3098	546	1254	1000
Number of animals only with known sire	258	88	98	6
Number of animals only with known dam	26	15	8	2
Number of animals with no known parents	1579	617	421	59
**Reproductive Parameter**	**Historical Period**	**2008–2012 Period**	**2013–2017 Period**	**2018–2022 Period**
Average number of calves per sire	7.14	7.54	7.87	5.80
Maximum number of calves per sire	113	40	35	24
Average number of calves per dam	1.96	2.13	2.30	1.86
Maximum number of calves per dam	30	13	30	8

* The historical-period-related figures included individuals/data from before 2008.

**Table 2:** Pedigree-completeness-derived parameters in Ecuadorian Charolais cattle from 2008 to 2022.

Pedigree Completeness	Historical Period * (n = 4961)	2008–2012 Period (n = 1266)	2013–2017 Period (n = 1781)	2018–2022 Period (n = 1067)
First generation (%)	65.35	47.20	73.39	94.10
Second generation (%)	47.84	28.46	53.54	84.70
Third generation (%)	33.23	16.24	36.34	68.38
Fourth generation (%)	18.77	5.96	18.90	46.45
Fifth generation (%)	9.25	2.20	7.75	26.85
Average GMax-FF	7.83	6.00	6.86	8.36
Average GCom-FF	2.05	1.97	2.02	2.07
Average GEqu-FF	3.76	3.01	3.49	3.93
Average GMax-PB	3.57	2.23	4.05	6.38
Average GCom-PB	0.75	0.46	0.84	1.39
Average GEqu-PB	1.49	0.92	1.68	2.75
Number of generations	11	9	10	11

GMax: maximum number of generations; GenCom: complete number of generations; GEqu: equivalent number of generations; FF: Full French; PB: Purebred. * The historical-period-related figures included individuals/data from before 2008.

**Table 3:** Gene origin probability and genetic-diversity-derived parameters in Ecuadorian Charolais cattle from 2008 to 2022.

Gene-Origin/Genetic Diversity Parameters	Reference Population (n = 3068)	2008–2012 Period (n = 549)	2013–2017 Period (n = 1253)	2018–2022 Period (n = 970)
Historical population * (n)	4937	1271	1777	1041
Number of ancestors contributing to the reference population (n)	1063	372	646	361
Number of founders contributing to the reference population (n)	1146	539	847	544
Effective number of founders (fe)	207.72	168.92	189.65	131.81
Effective number of non-founders (Nenf)	91.94	95.96	90.64	37.23
Founder genome equivalents (fg)	63.73	61.19	61.33	29.03
Effective number of ancestors (fa)	97	78	90	43
fa/fe ratio	0.47	0.46	0.47	0.33
fg/fa ratio	0.66	0.78	0.68	0.68
fg/fe ratio	0.31	0.36	0.32	0.22
Number of ancestors to explain:				
25% of gene pool	10	8	10	5
50% of gene pool	41	40	37	18
75% of gene pool	203	146	161	58
GD	99.22	99.18	99.18	98.28
1-GD (GD loss)	0.78	0.82	0.82	1.72
DG*	99.76	99.70	99.74	99.62
Proportion of unequal contributions of the founders in GD loss (%)	0.24	0.30	0.26	0.38
Proportion of random genetic drift and bottle necks in GD loss (%)	0.78	0.82	0.82	1.72

GD: Genetic diversity. The probability of gene origin given by the effective number of founders. DG*: Genetic diversity in the reference population considered to compute the genetic diversity loss due to the unequal contribution of founders; effective numbers of founders (fe) and founder genome equivalents (fg) are also shown. * The historical-period-related figures included individuals/data from before 2008.

## Data Availability

All data are contained within the article.
